# Synthesis of rhamnolipid biosurfactant and mode of hexadecane uptake by *Pseudomonas *species

**DOI:** 10.1186/1475-2859-8-16

**Published:** 2009-03-11

**Authors:** Swaranjit Singh Cameotra, Pooja Singh

**Affiliations:** 1 Institute of Microbial Technology, Sector 39A, Chandigarh, 160036, India

## Abstract

**Background:**

Microorganisms have devised ways by which they increase the bioavailability of many water immiscible substrates whose degradation rates are limited by their low water solubility. Hexadecane is one such water immiscible hydrocarbon substrate which forms an important constituent of oil. One major mechanism employed by hydrocarbon degrading organisms to utilize such substrates is the production of biosurfactants. However, much of the overall mechanism by which such organisms utilize hydrocarbon substrate still remains a mystery.

**Results:**

With an aim to gain more insight into hydrocarbon uptake mechanism, an efficient biosurfactant producing and n-hexadecane utilizing *Pseudomonas sp *was isolated from oil contaminated soil which was found to produce rhamnolipid type of biosurfactant containing a total of 13 congeners. Biosurfactant action brought about the dispersion of hexadecane to droplets smaller than 0.22 μm increasing the availability of the hydrocarbon to the degrading organism. Involvement of biosurfactant was further confirmed by electron microscopic studies. Biosurfactant formed an emulsion with hexadecane thereby facilitating increased contact between hydrocarbon and the degrading bacteria. Interestingly, it was observed that "internalization" of "biosurfactant layered hydrocarbon droplet" was taking place suggesting a mechanism similar in appearance to active pinocytosis, a fact not earlier visually reported in bacterial systems for hydrocarbon uptake.

**Conclusion:**

This study throws more light on the uptake mechanism of hydrocarbon by *Pseudomonas aeruginosa*. We report here a new and exciting line of research for hydrocarbon uptake involving internalization of biosurfactant covered hydrocarbon inside cell for subsequent breakdown.

## Background

Pollution due to petroleum oil is even now a prevalent ecological hazard and hence microbial degradation of hydrocarbons remains as topical an issue as before. A common feature of all the fractions of crude oil is their low water solubility and this poses special problems for those microorganisms capable of utilizing such water immiscible substrates as source of carbon and energy. The first step in the process is the transport of the hydrocarbon from oil phase to the cell surface in some way so as to achieve effective cell surface contact and ultimately efficient transportation across cell membrane, or in other words uptake/intake. Although much work has been done in this field, the mechanism of n-alkane transport to bacterial cell and its subsequent assimilation inside cells still remains obscure.

Two types of hydrocarbon interaction during biodegradation has been described earlier by Kirschner [1]: adhesion to oil, and a hypothesized pseudosolubilization in which the hydrocarbon degrading bacteria assimilate small droplets of emulsified oil. Reports are available in favor of the first theory where microbial cells attach to the surface of hydrocarbon drops much smaller than the cells and substrate uptake presumably takes place through diffusion or active transport at the point of contact [2,3]. The hypothesized emulsification and incorporation of submicron droplets of oil [4,5] is analogous to the pseudosolubilization of oil hypothesized by Kirschner. Similar theory was explained by Singh and Desai [6]. They referred to two modes of initial interaction of hydrocarbon with the microbial cells: (a) Direct contact of microorganisms with insoluble substrate (unmediated interaction) or (b) by the contact through a mediator (mediated interaction. The scale tilts in the favor of the latter theory involving the role of mediators or extracellular solubilizing factor.

Several investigators have shown the presence of emulsifiers in the culture broth during the growth of microorganisms on hydrocarbons and its effect, due to solubilization/emulsification, on hydrocarbon uptake [7-9]. Biosurfactants are present in various pools inside cells: as intracellular molecules, extracellularly secreted compounds or as compounds located at the cell surface [10]. Together they have been known to enhance degradation by alteration in cell hydrophobicity and enhancement of dispersion of water immiscible compounds [11-13]. *P. aeruginosa *strains are known to produce rhamnolipid type of biosurfactant, reported to be a mixture of mainly mono and dirhamnolipids. Addition of rhamnolipids to pure cultures or in soil systems has been shown to enhance biodegradation of a number of hydrocarbons including hexadecane [14-19]. Thus a clear correlation exists between surface active agent production and alkane utilization by the degrading organism. However, Different modes of uptake have been proposed for different microorganisms for the growth on hydrocarbons, sometimes more than one mechanism occurring simultaneously [20]. Hence, no conclusive picture has come out of the various studies till now.

In the present study, we have tried to look into the matter of hydrocarbon uptake by a hydrocarbon degrading *Pseudomonas *strain isolated from oil-contaminated soil. Characterization of biosurfactant produced was done and its role in hydrocarbon uptake studied. Electron Microscopic studies were done extensively to elucidate the mechanism(s) adopted to uptake water insoluble hydrocarbon into bacterial cell and some interesting results were obtained which would go on to consolidate the theories of hydrocarbon uptake by bacterial cells.

## Methods

### Chemicals and media

All the chemicals used were of the highest purity grade. Minimal media used in the study contained 28 mM disodium hydrogen phosphate, 14.7 mM potassium dihydrogen orthophosphate, 6.1 mM ammonium sulphate, 6.6 mM magnesium sulphate and 1ml of trace element solution (TES) per liter. TES contained 1.30 mM Al(OH)_3_, 0.22 mM SnCl_2_.2H_2_O, 0.30 mM KI, 1.2 mM LiCl, 0.42 mM MgSO_4_.4H_2_O, 8.10 mM H_3_BO_3_, 0.35 mM ZnSO_4_.7H_2_O, 0.42 mM CoCl_2_.6H_2_O, 0.38 mM NiSO_4_.6H_2_O, 0.24 mM BaCl_2 _and 0.35 mM (NH_4_)I. n-hexadecane used as the test hydrocarbon was obtained from Sigma.

### Isolation and biodegradation studies

The organism used in the study was isolated from soil contaminated with crude oil sludge. In all the growth studies, temperature of incubation was 37°C and aeration was maintained by agitation on a rotary shaker (210 rpm). A bacterial isolate designated as SSC2 was isolated from oil contaminated soil by serial dilution plating method on Nutrient Agar media as well as on minimal medium with 2% (v/v) n-hexadecane as the sole source of carbon and energy. Screening was done by (i) monitoring change in dry biomass upon different times of growth on n- hexadecane and (ii) residual hydrocarbon (n-hexadecane) analysis. Degradation of n-hexadecane was monitored by Gas-Chromatographic analysis of residual hydrocarbon extracted using hexane as the solvent. For GC analysis, a Hewlett Packard (series II Gas 5890) instrument with cross-linked methyl silicone gum capillary column of 25 mm × 0.2 mm × 0.33 μm film thickness and equipped with FID was used. Oven temperature was standardized at 300°C with injector and detector at 350°C.

### Characterization of the selected isolate

SSC2 was characterized using various morphological and biochemical tests and confirmed by whole cell fatty acid profile analysis by GC- Microbial Identification System (MIS) procured from Hewlett Packard. For identification by morphological and biochemical means, Bergy's Manual of Systemic Bacteriology, Vol. 1 and the Manual for the Identification of Medical Bacteria by Cowan & Steel (Second Edition, Cambridge University press) was used. Final confirmation by 16srDNA sequence analyses was also done.

### Pseudosolubilization studies

SSC2 was studied for the production of biosurfactant by monitoring the lowering of surface tension of the cell free broth after different time intervals. Surface Tension was measured using a DuNouy Tensiometer (CSC NO. 70535, CSC, Albany, NY, USA) and surface tension of broth was directly recorded in dynes cm^-1^. Cell free broth of different day's growth was used to study the presence of different sized hydrocarbon droplets in the culture media. SSC2 was grown in minimal media with n-hexadecane as the sole carbon source. Cell free broth was filtered through 0.45 and 0.22 μm Millipore membrane filters. Residual hexadecane was extracted from the filtrates using hexane and analyzed by GC using the same parameters mentioned earlier. Another bacterial soil isolate SSC1, a not so efficient hydrocarbon degrader and biosurfactant producer, was also processed in similar way for studying the formation of varying sized droplets. Another unfiltered parallel set for both the strains was used for total residual hydrocarbon analyses at different times and used for percent hydrocarbon calculation after filtration.

### Electron Microscopy studies for hydrocarbon uptake

Scanning Electron Microscopy (SEM) was performed on SSC2 grown in minimal medium with 2% n-hexadecane as carbon source. After 2, 4 and 6 days of growth, cells were spinned down and washed thrice with PBS (phosphate buffer saline, pH 7.2) before being fixed in 2.5% glutaraldehyde made in PBS with overnight incubation at 4°C. After three successive gentle washings in PBS, cells were suspended in minimum amount of PBS and layered onto 1mg/ml polylysine coated cover slips. These were then dehydrated through a series of alcohol dehydration steps (30, 50, 70, 90 and 100%) and finally layered with t-butyl alcohol for freeze-drying. Freeze dried samples were viewed under a Jeol Scanning Electron Microscope-6100. Cells grown for 48–60 hours on 2% (w/v) glucose in minimal medium were taken as control and processed similarly.

For ultrastructural studies, 2, 4 and 6 day old samples were washed in 10 mM phosphate buffer (pH7.2), fixed in 2.5% gluteraldehyde and post fixed in 1% osmium tetraoxide after successive washings in 10 mM sodium phosphate buffer pH 7.4. After several washes in the same buffer, cells were dehydrated in graded acetone solutions and embedded in CY 212 araldite, along with Dodecenyl Succinic Anhydride (DDSA) as hardner, and tri(dimethylaminomethyl) phenol (DMP-30) as catalyst. Ultrathin sections of 60–80 nm thickness were cut using an ultracut E (Reichert Jung) ultramicrotome and the sections were stained in alcoholic uranyl acetate (10 min) and subsequently in lead acetate (10 min) before examining the grids in either CM-10 Phillips Transmission Electron Microscope (TEM) operated at 80kV or Morgagni 268 D Transmission Electron Microscope FEI operated at 80kV which gave better resolution at low magnification. Cells grown for 48–60 hours on 2% (w/v) glucose in minimal medium were taken as control and processed similarly.

### Analyses of intracellular hydrocarbon

To analyze the nature of numerous intracytoplasmic inclusions observed in hydrocarbon grown cells 2, 3 and 5 day n-hexadecane grown cells were harvested and washed with phosphate buffer (10 mM; pH 7.0) and with 50 mM tris (hydroxymethyl) aminomethane chloride buffer (pH 7.8) to remove all the adhering extracellular hydrocarbons [21]. 2 and 3 day old SSC2 cells grown on 2% glucose were taken as control and processed similarly. Intracytoplasmic hydrocarbon extraction was done by chloroform using lyophilized cells [22]. 2 μl of the extracted organic phase was analyzed using Shimadzu GC-17A equipped with Flame Ionization Detector and a DB1 column 30 m × 0.25 mm in size. Injector temperature was 250°C with the detector at 300°C. Oven temperature was kept at 100°C for 2 min and increased to 300°C at a rate of 20°C per min. and kept at 300°C for 20 min.

## Results and discussion

### Isolation and characterization of the hydrocarbon degrading strain

**Figure 1:**
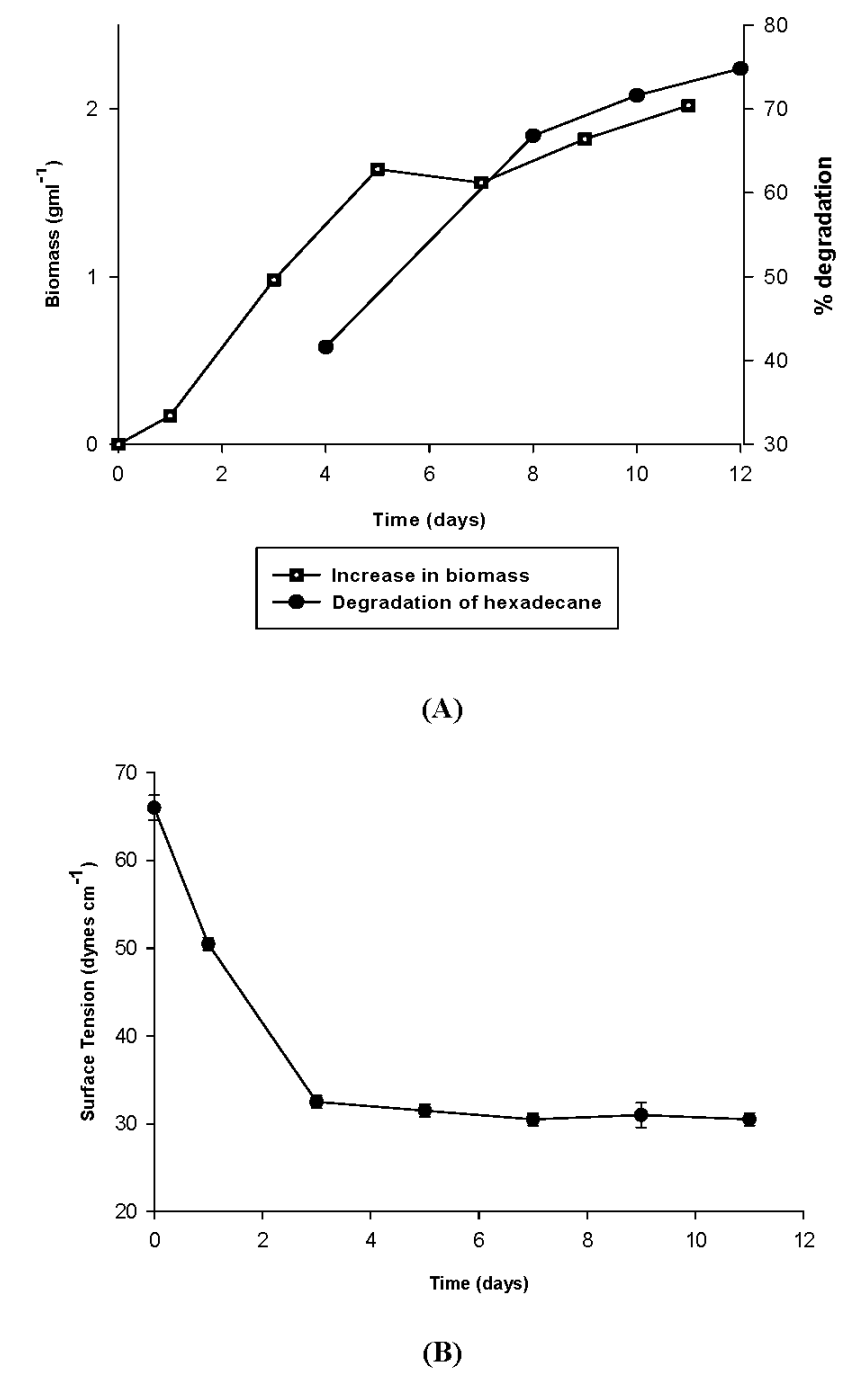
**(A): Change in biomass (growth) and degradation of hydrocarbon (n-hexadecane) by SSC2**. (B): Change in surface tension of culture broth upon growth of SSC2 (indication of biosurfactant production).

Out of the many isolates obtained from oil contaminated soil, bacterial isolate SSC2 was finally selected for hydrocarbon uptake studies. Morphological and biochemical tests showed SSC2 to be a motile and aerobic gram-negative rod exhibiting fluorescence under UV light. The organism was able to grow at 42°C but growth at 4°C was absent. It gave positive reaction for cytochrome oxidase, catalase and also arginine dehydrolase, which is a characteristic reaction of *Pseudomonas aeruginosa*. All these and other biochemical tests (e.g. starch, casein and gelatin hydrolysis, acid production profiles etc.) suggested the isolate to be *Pseudomonas aeruginosa*, which was confirmed by fatty acid analysis using Microbial Identification System where the isolate gave a similarity index of 0.918 with 100% peaks named. 16srDNA sequence analyses confirmed the isolate to be *Pseudomonas aeruginosa *with 99% identity. Both growth and degradation were fastest after 3 days and became stable after around 11 days of growth. Growth of SSC2 started at a rate of 0.8 g l^-1 ^per hr and a maximum of 2.1 g l^-1 ^biomass was obtained after 11 days of growth. A degradation of around 70% hexadecane was observed in 7 days time after which no significant increase in degradation was observed, although slow degradation did continue to upto 75% till the time of analyses (Figure 1A). SSC2 was found to lower the surface tension of culture broth from 65 dynes cm^-1 ^to 30 dynes cm^-1 ^in 3 days time which corresponded to a simultaneous increase in growth and degradation as mentioned earlier (Figure 1B). Biosurfactant production is a commonly observed phenomenon upon growth of the organism on hydrophobic substrates and reduction in surface tension is indicative of efficient biosurfactant production [23].

### Production of biosurfactant and pseudosolubilization of hydrocarbons

**Figure 2:**
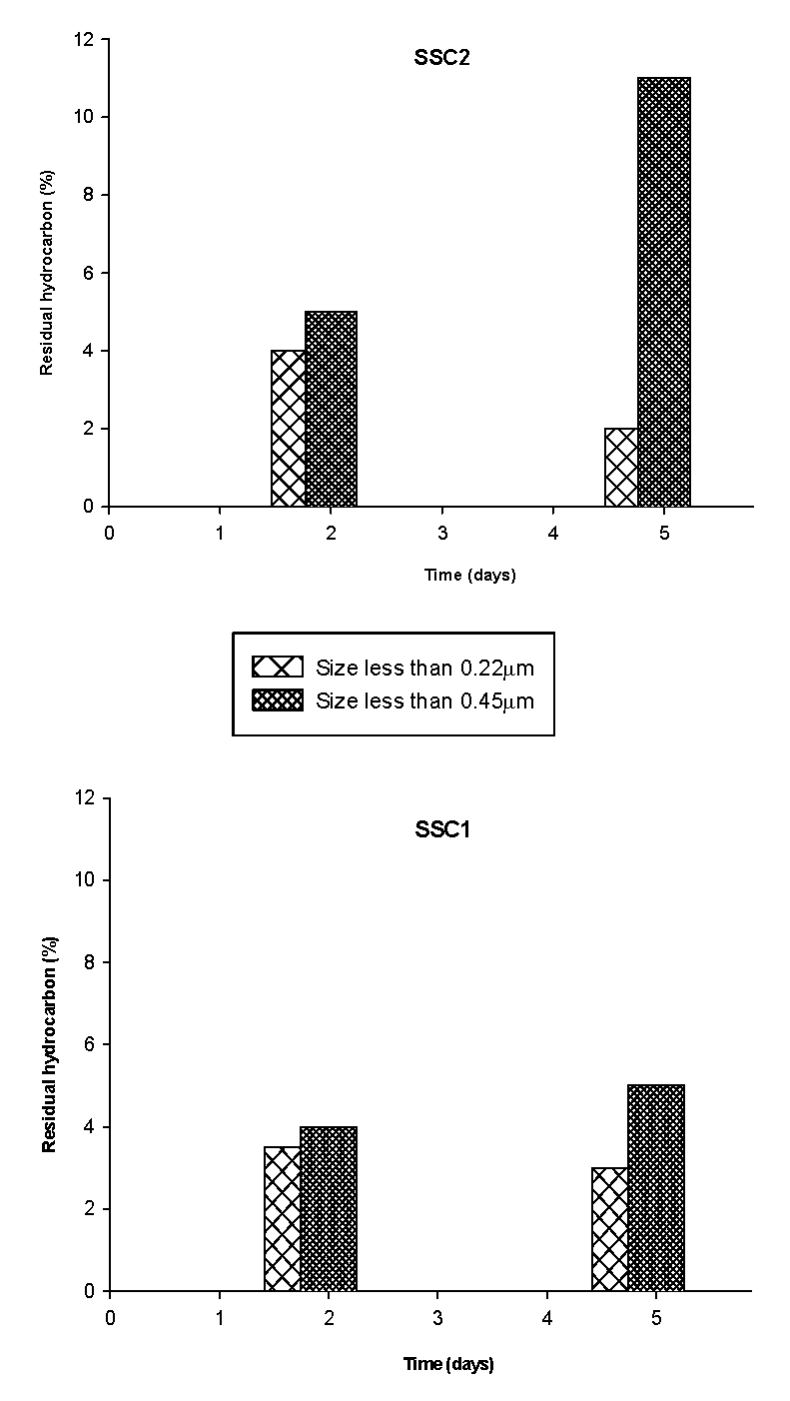
**Pseudosolubilization/dispersion of hexadecane indicated by formation of small sized droplets**.

Analyses of different sized hydrocarbons in the culture broth after 2 days of growth revealed the presence of droplets smaller than 0.22 μm and also bigger droplets of size in between 0.45 and 0.22 μm. After 5 days of growth, droplets of size in between 0.22 to 0.45 μm increased markedly, indicative of enhanced dispersion of the hydrophobic substrate by the bacteria (Figure 2). On the other hand there was a decrease in the amount of droplets smaller than 0.22 μm after the same time indicating preferential microbial utilization of smaller droplets as they were formed after dispersion. Amount of dispersed substrate by SSC1, a poor biosurfactant producer, was much less as compared to that by SSC2, a good biosurfactant producer, even after 5 days of growth. Hydrocarbon solubilizing factors have been earlier reported to be produced by microorganisms growing on liquid hydrocarbon and have been shown to enhance hydrocarbon degradation rate by formation of hydrocarbon in water emulsion and also microemulsions/pseudosolubilization [24]. Hence for our strain, dispersion was an important phenomenon for efficient degradation. This observation coupled with the observation that SSC2 was a very efficient biosurfactant producer, signifies the role of biosurfactant in hydrocarbon degradation. Biosurfactant produced by SSC2 was analyzed to be a rhamnolipid mixture containing upto 13 different congeners including mono and dirhamnolipids [25].

### Electron Microscopic studies

**Figure 3:**
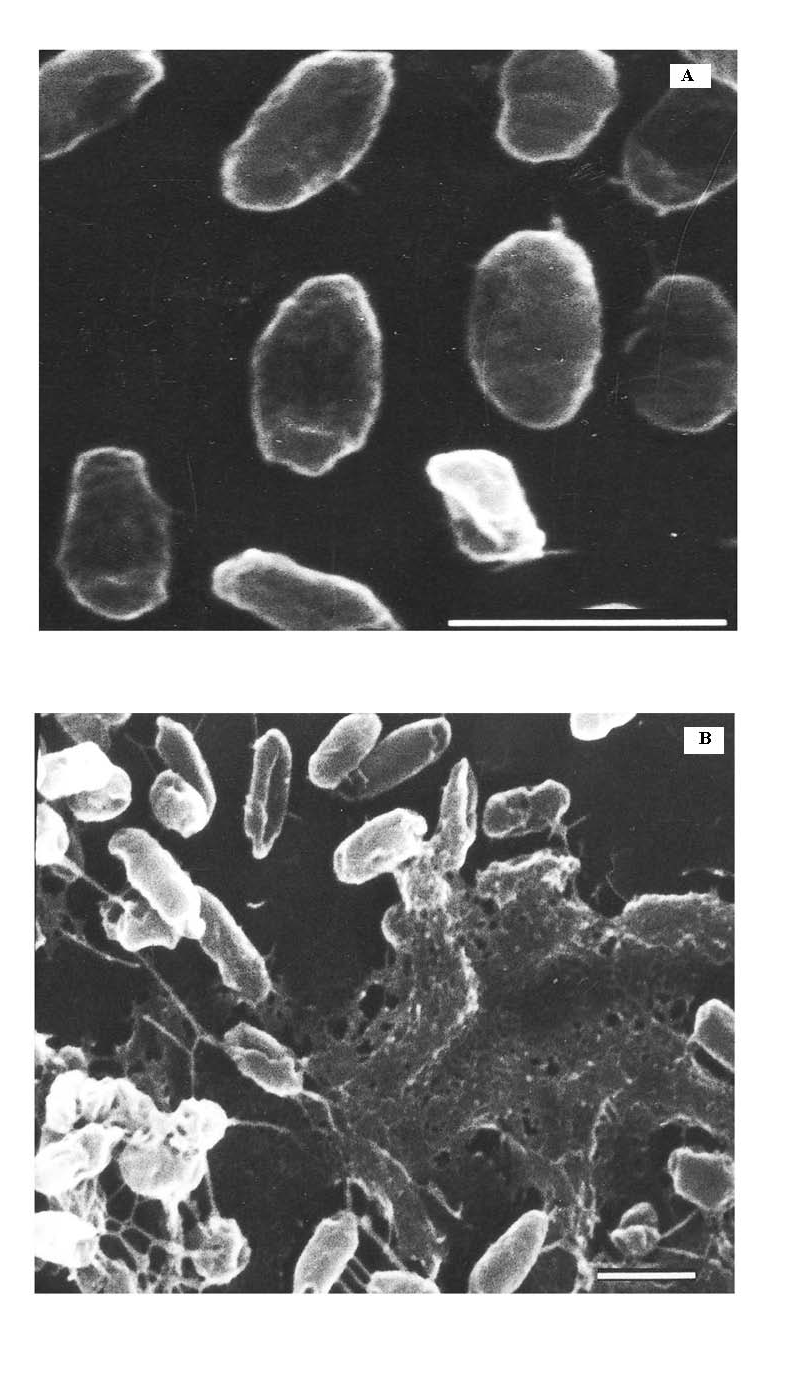
**Scanning Electron Microscopy of SSC2 grown on**. (A) 2% glucose, and (B) 2% n-hexadecane. Bar is equivalent to a length of 1 μm.

A number of cytological and morphological changes have been observed in microbial cells as a result of presence of hydrocarbons but most of the work has been done on yeasts especially *Candida *sp. due to the convenience of study [26-28]. Limited literature is also there for *Acinetobacter *species [29,30]. Not much report is there on changes in *Pseudomonas *as a result of growth on hydrocarbons and the mode of substrate uptake by the same. Scanning Electron Microscopic studies on SSC2 grown on glucose and n-hexadecane revealed many changes in the bacterial cells (Figure 3). Cells grown on hydrocarbon appeared longer and large amount of "web like projections" were observed. Cells were found to be kind of connected to each other by means of numerous fiber like projections and were found to be concentrated at areas of network formed by the extracellular secretion (Figure 3B). All these were absent when they were grown on glucose as carbon source (Figure 3A). The fiber like network attracting bacterial cells observed in SEM could be a form of alkane and surfactant complex. This complex could be a way by which hydrocarbon is transported to cell surface for further uptake and also a kind of anchorage site for cells to feed on hydrocarbons.

**Figure 4:**
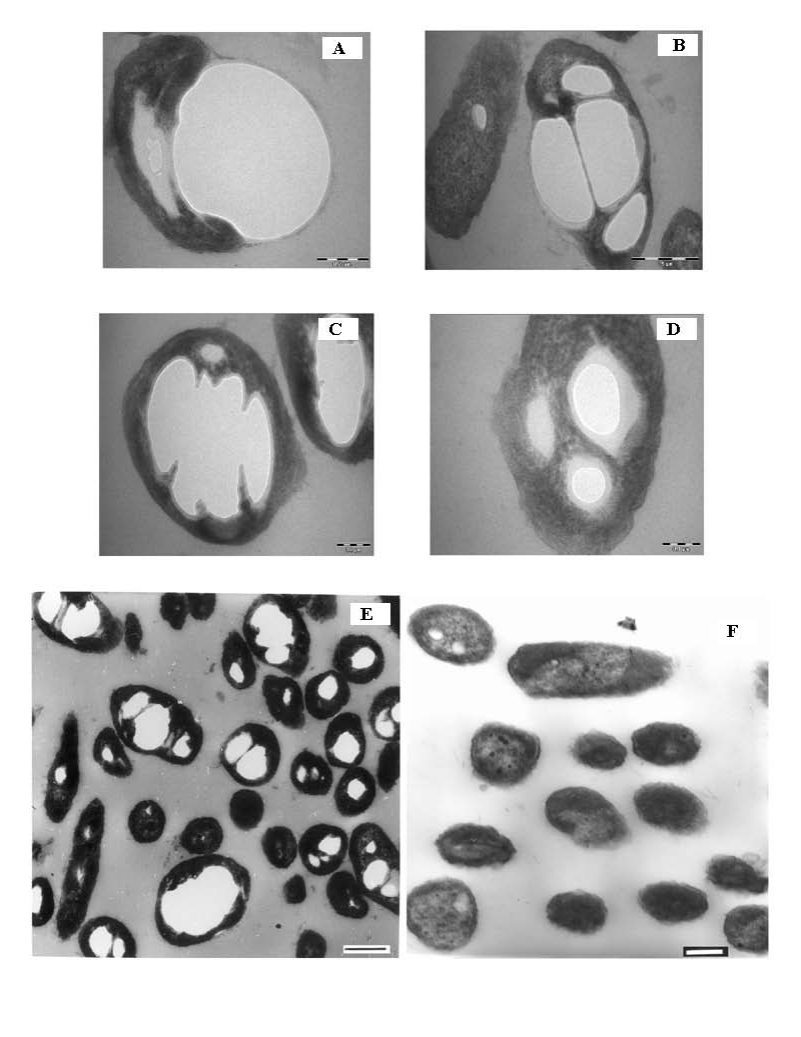
**Transmission Electron Microscopy of SSC2 grown on 2% n-hexadecane (5A-E) and 2% glucose (5F)**. Figures 4 (A-D) were from Morgagni TEM. Figures 5(5) and 5(F) were from CM 10 Phillips TEM. Bar in different figures stand as follows: (A): 0.2 μm, (B): 0.5 μm, (C): 0.2 μm, (D): 0.1 μm, (E): 0.3 μm (F): 0.2 μm.

Ultra structural studies revealed the presence of numerous electron transparent inclusions within hydrocarbon grown cells (Figure 4). Cells were found to attach to large hydrocarbon droplets, however smaller droplets attached to and present within the cells were also observed. Cells were found to kind of "engulf" hydrocarbon droplets of volumes comparable to cell size or even larger (Figure 4A). Droplets after the process of being "internalized" had an electron dense fibrous layer around them on the areas exposed outside. This layer was found to be continuous with the surface of the cell (Figure 4B). Once inside, the hydrocarbon droplet appeared to be broken down by means of internal cellular projections possibly to increase the surface area for further hexadecane metabolism (Figure 4C). Cells appeared swollen at the point of internalization of the droplet (Figure 4D). Numerous cells with large amount of hydrocarbon droplets inside them were observed (Figure 4E). None of these were observed in glucose grown cells (Figure 4F).

**Figure 5:**
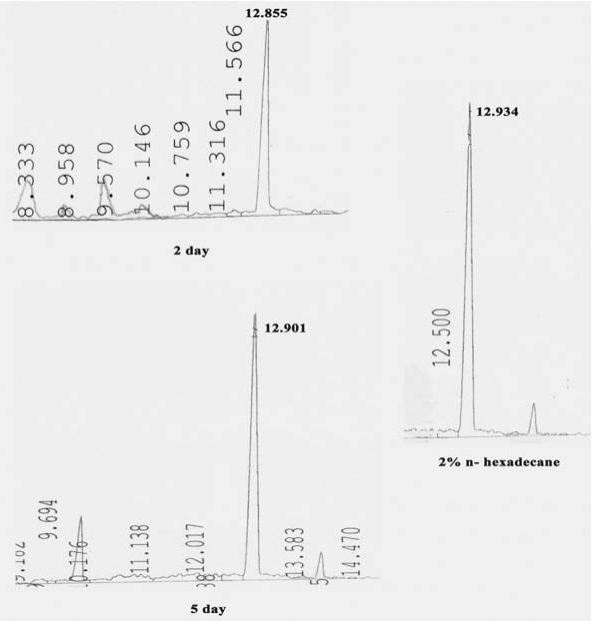
**GC analyses of extracted intracellular hydrocarbon from cells of SSC2 at different times of growth**. Right panel shows 2% n-hexadecane as standard. Peak at Retention time around 12.9 corresponds to that of n-hexadecane (unmodified substrate).

GC analyses of intracellular hydrocarbon from cells grown on hexadecane revealed the presence of unmodified hexadecane in the numerous inclusions (Figure 5). A number of peaks were present in the chloroform extract of cells grown for different days on n-hexadecane as well as glucose but a peak at RT of 12.5 minutes (average) was observed to be present in cells grown on hydrocarbon while it was absent in the glucose grown cells. This peak was found to be n-hexadecane as was evident after GC analyses of 2% hexane solution of n-hexadecane. Earlier, a comparative analysis of the ultrastructural changes upon hydrocarbon uptake was done in a variety of hydrocarbon oxidizing microorganisms and in almost all the cases, growth in hydrocarbon was characterized by the presence of intracellular electron-transparent inclusions which appeared to be membrane bound and upon analyses were found to be respective hydrocarbon growth substrate [31]. In the present study too, presence of unmodified hexadecane was observed in cells grown at different time intervals and they appeared membrane bound.

In the present study, many mechanisms proposed for hydrocarbon uptake were found to be playing a joint role in facilitating n-hexadecane uptake:

Direct contact of cells with large hydrocarbon droplets.

Interaction of cells with "solubilized/pseudosolubilized" or "accommodated" hydrocarbon droplets of size comparable to the cell size (enhanced dispersion and pseudosolubilization of hydrophobic substrate being the process facilitating enhanced contact for uptake of the substrate)

Uptake of hydrocarbon by internalization of biosurfactant covered substrate.

## Conclusion

A process similar in appearance to active pinocytosis was observed in the present electron microscopic studies, for the internalization of biosurfactant covered hydrocarbon droplets of large sizes. Earlier this theory has been proposed in yeasts where vesicle formation at the cell surface was suggested to arise by pinocytosis of alkane [32]. No occurrence/report of a similar process is there for bacteria. In one of the closest studies on different mechanisms of hydrocarbon uptake by bacteria, it was proposed that biosurfactant only act to enhance the process of hexadecane uptake and are not required for the process to take place [33]. However, our electron microscopic findings question this hypothesis and we propose the involvement of biosurfactant to be extended beyond just enhancement of the bioavailability of the substrate. The mechanism reported here is similar to the one proposed by Ratledge [34] whereby surfactants act to enhance the process of pseudosolubilization/emulsification thereby increasing the availability of hydrocarbon to cells. However, the role of biosurfactant does not end here. Hydrocarbon droplet enclosed within a surfactant layer is the form in which cells seem to take up the hydrocarbon. As proposed earlier by Ratledge [34], the requirement of energy for the uptake process would be divided between pinocytosis and movement of the alkane free surfactant from inside the cell (through the cell envelope) to the outside. Another factor could be utilization of the internalized biosurfactant for the formation of membranes around the hydrocarbon droplet and its further partitioning into smaller compartments for increased surface area for metabolism. The entire process however, is a combination of predominantly two modes of uptake proposed earlier *viz*. uptake by direct contact and pseudosolubilization of hydrocarbon to microdroplets, both facilitated by biosurfactants and occurring simultaneously to enhance and facilitate hexadecane uptake.

## Competing interests

The authors declare that they have no competing interests.

## Authors' contributions

PS carried out this research work for her Ph. D. degree and SSC supervised the study, conceived of the study, and participated in its design and coordination. All authors read and approved the final manuscript.

## References

[B1] Kirschner ZI, Rosenberg E, Gutnick D (1980). Incorporation of P^32 ^and growth of Pseudomonad UP-2 on n-tetracosane. Appl Environ Microbiol.

[B2] Kennedy RS, Finnerty WR, Sudarshan K, Young RA (1975). Microbial assimilation of hydrocarbons I. The fine structure of a hydrocarbon oxidizing *Acinetobacter *species. Arch Microbiol.

[B3] Rosenberg M, Rosenberg E (1981). Role of adherence in growth of *A. calcoaceticus *RAG-1 on hexadecane. J Bacteriol.

[B4] Rosenberg C, Zukerberg A, Rubinovitz C, Gutnick DL (1979). Emulsifier of *Arthrobacter *RAG-1: isolation and emulsifying properties. Appl Environ Microbiol.

[B5] Reddy PG, Singh HD, Roy PK, Baruah JN (1982). Predominant role of hydrocarbon solubilization in the microbial uptake of hydrocarbons. Biotechnol Bioengg.

[B6] Singh M, Desai JD (1986). Uptake of water insoluble substrates by microorganisms. J Sci Indus Res.

[B7] Cameotra SS, Singh HD, Baruah JN (1984). Demonstration of extracellular alkane solubilization factor produced by (*Endomycopsis lipolytica*) YM. Biotechnol Bioengg.

[B8] Southam G, Whitney M, Knickerbocker C (2001). Structural characterization of the hydrocarbon degrading bacteria-oil interface: implications for bioremediation. Biodeterio Biodegrad.

[B9] Cubitto MA, Moran AC, Commendatore M, Chiarello MN, Baldini MD, Sineriz F (2004). Effects of *Bacillus subtilis *O9 biosurfactant on the bioremediation of crude oil-polluted soils. Biodegrad.

[B10] Prabhu Y, Phale P (2003). SB PP2 novel metabolic pathway, role of biosurfactant and cell surface hydrophobicity in hydrocarbon assimilation. Appl Microbiol Biotechnol.

[B11] Zang Y, Miller RM (1992). Enhanced octadecane dispersion and biodegradation by a *Pseudomonas *rhamnolipid surfactant (biosurfactant). Appl Environ Microbiol.

[B12] Zang Y, Miller RM (1994). Effect of a *Pseudomonas *rhamnolipid biosurfactant on cell hydrophobicity and biodegradation of octadecane. Appl Environ Microbiol.

[B13] Patricia B, Jean-Claude B (1999). Involvement of bioemulsifier in heptadecane uptake in *Pseudomonas nautical*. Chemosphere.

[B14] Oberbremer A, Muller-Hurtig R, Wagner F (1990). Effect of the addition of microbial surfactants on hydrocarbon degradation in a soil population in a stirred reactor. Appl Microbiol Biotechnol.

[B15] Herman DC, Lenhard RJ, Miller RM (1997). Formation and removal of hydrocarbon residual in porous media: effects of bacterial biomass and biosurfactant. Environ Sci technol.

[B16] Herman DC, Zhang Y, Miller RM (1997). Rhamnolipid (biosurfactant) effects on cell aggregation and biodegradation of residual hexadecane under saturated flow conditions. Appl Environ Microbiol.

[B17] Sandrin TR, Chech AM, Maier RM (2000). Protective effect of a rhamnolipid biosurfactant on naphthalene biodegradation in the presence of cadmium. Appl Environ Microbiol.

[B18] Maslin P, Maier RM (2000). Rhamnolipid enhanced mineralization of phenanthrene by indigenous microbial populations in organic-metal contaminated soils. Bioremed J.

[B19] Noordman WH, Wachter JH, de Boer GJ, Janssen DB (2002). The enhancement by surfactants of hexadecane degradation by *Pseudomonas aeruginosa *varies with substrate availability. J Biotechnol.

[B20] Goswami P, Singh HD (1991). Different modes of uptake by two *Pseudomonas *species. Biotechnol Bioengg.

[B21] Scott CCL, Finnerty WR (1976). A comparative analysis of the ultrastructure of hydrocarbon-oxidizing microorganisms. Gen J Microbiol.

[B22] Brandl H, Gross RA, Lenz RW, Fuller RC (1988). *Pseudomonas oleovorans *as a source of poly (β-hydroxyalkanoates) for potential applications as biodegradable polyesters. Appl Environ Microbiol.

[B23] Pruthi V, Cameotra SS (1995). Rapid method for monitoring maximum biosurfactant obtained by acetone precipitation. Biotechnol Techniq.

[B24] Roy PK, Singh HD, Bhagat SD, Baruah JN (1979). Characterization of hydrocarbon emulsification and solubilization occurring during the growth of *Endomycopsis lipolytica *on hydrocarbons. Biotechnol Bioeng.

[B25] Cameotra SS, Singh P (2008). Bioremediation of oil sludge using crude biosurfactants. Internat Biodet Biorem.

[B26] Kappeli O, Fiechter A (1977). Component from the cell surface of hydrocarbon utilizing yeast *Candida tropicalis *with possible relation to hydrocarbon transport. J Bacteriol.

[B27] Kappeli O, Muller M, Fiechter A (1978). Chemical and structural alterations at the cell surface of *Candida tropicalis *induced by hydrocarbon substrate. J Bacteriol.

[B28] Kappeli O, Fiechter A (1981). Properties of hexadecane uptake by *Candida tropicalis*. Curr Microbiol.

[B29] Kennedy RS, Finnerty WR (1975). Microbial assimilation of hydrocarbons. II. Intracytoplasmic membrane induction in *Acin etobacter *sp. Arch Microbiol.

[B30] Kappeli O, Finnerty WR (1979). Partition of alkane by an extracellular vesicle derived from hexadecane grown *Acinetobacter*. J Bacteriol.

[B31] Scott CCL, Finnerty WR (1976). Characterization of intracytoplasmic hydrocarbon inclusions from the hydrocarbon-oxidizing *Acinetobacter *species HO1-N. J Bacteriol.

[B32] Meisel MN, Medvedeva GA, Kozlova TM, Domoshnikova NA, Zaikina AI, Fedoseeva GE, Suomalainen H (1973). Regularities of penetration into yeast cells of higher fatty acids and hydrocarbons, their intracellular migration and concentration. Proceedings of the 3rd International Specialized Symposium on yeasts.

[B33] Beal R, Betts WB (2000). Role of rhamnolipid biosurfactants in the uptake and mineralization of hexadecane in *Pseudomonas aeruginosa*. J Appl Microbiol.

[B34] Ratledge C (1992). Chemistry of aliphatic hydrocarbons and degradation. Latin American Biodeterioration Symposium.

